# Case report of hypercalcemia-related kidney complications after discontinuation of denosumab

**DOI:** 10.3389/fped.2025.1583240

**Published:** 2025-06-06

**Authors:** Gerrit van den Berg, Marc R. Lilien, Rutger R. G. Knops, Robert J. J. van Es, Atty T. H. van Dijk, Mandy G. Keijzer-Veen

**Affiliations:** ^1^ Department of Pediatric Nephrology, Wilhelmina Children’s Hospital, University Medical Center Utrecht, Utrecht, Netherlands; ^2^ Princess Máxima Center for Pediatric Oncology, Utrecht, Netherlands; ^3^ Department of Maxillofacial Surgery and Head and Neck Surgical Oncology, University Medical Center Utrecht, Utrecht, Netherlands; ^4^ Expert Center for Skeletal Dysplasia, Wilhelmina Children’s Hospital, University Medical Center Utrecht, Utrecht, Netherlands

**Keywords:** denosumab, acute kidney injury, hypercalcemia, case report, pediatric nephrology

## Abstract

**Background:**

The use of the osteoclastogenesis inhibitor denosumab is increasing in pediatrics, especially in the treatment of giant cell tumor or granuloma of bone or jaw, aneurysmal bone cyst, and other rare bone disorders. Particularly in pediatric patients, adverse kidney effects—such as acute kidney injury (AKI), hypertension, and nephrocalcinosis—are a significant concern that has received little attention.

**Case-diagnosis/treatment:**

In this report, we present three children who developed hypercalcemia-related AKI six months after discontinuation of denosumab treatment. Treatment of the hypercalcemia consisted of hyperhydration, and administration of furosemide, denosumab or bisphosphonate.

**Conclusions:**

Clinicians should be aware of the side effects of denosumab for at least seven months after discontinuation of denosumab. Early diagnosis and prompt management of hypercalcemia will result in recovery of AKI, however long-term consequences cannot be ruled out.

## Introduction

Denosumab is a humanized monoclonal antibody that targets the receptor activator of nuclear factor- κB ligand (RANKL). It is approved for the treatment of postmenopausal osteoporosis, glucocorticoid-induced osteoporosis, and cancer-related bone complications. In children, it is approved for the treatment of giant cell tumor of the bone (1). Currently, its therapeutic applications are expanding to aneurysmal bone cysts, central giant cell granulomas of the jaw bones, and other rare bone disorders. The mechanism of action of denosumab involves mimicking the binding of osteoprotegerin to RANKL, thereby suppressing osteoclast differentiation and activation, resulting in inhibition of bone resorption.

A significant adverse effect associated with discontinuation of denosumab is hypercalcemia, which can be assessed most accurately by measuring plasma ionized calcium, and if not available, serum total calcium adjusted for albumin concentration can be used. Hypercalcemia after discontinuation of denosumab is attributed to a rebound increase in osteoclastic bone resorption (2). Several recent reports have highlighted this phenomenon (3–6). Notably, an analysis of FDA reports elucidated the occurrence of rebound hypercalcemia following denosumab discontinuation. This analysis revealed a markedly higher incidence of this phenomenon in the pediatric population compared to adults. Specifically, the incidence rate was 28.5% in patients under 18 years of age, in contrast to 0.29% in patients 18 years and older. Among the pediatric cases, the onset of hypercalcemia was typically observed approximately 4 months after cessation of denosumab therapy (7). Notably, almost 20% (*n* = 10) of these patients also experienced acute kidney injury (AKI).

While denosumab is proven to be highly effective in suppressing bone resorption, and alleviating symptoms of various bone disorders, it has several side effects including kidney complications. In this context, we present three pediatric cases and a review of the literature of kidney complications due to hypercalcemia following denosumab discontinuation.

## Case description

**Figure 1:**
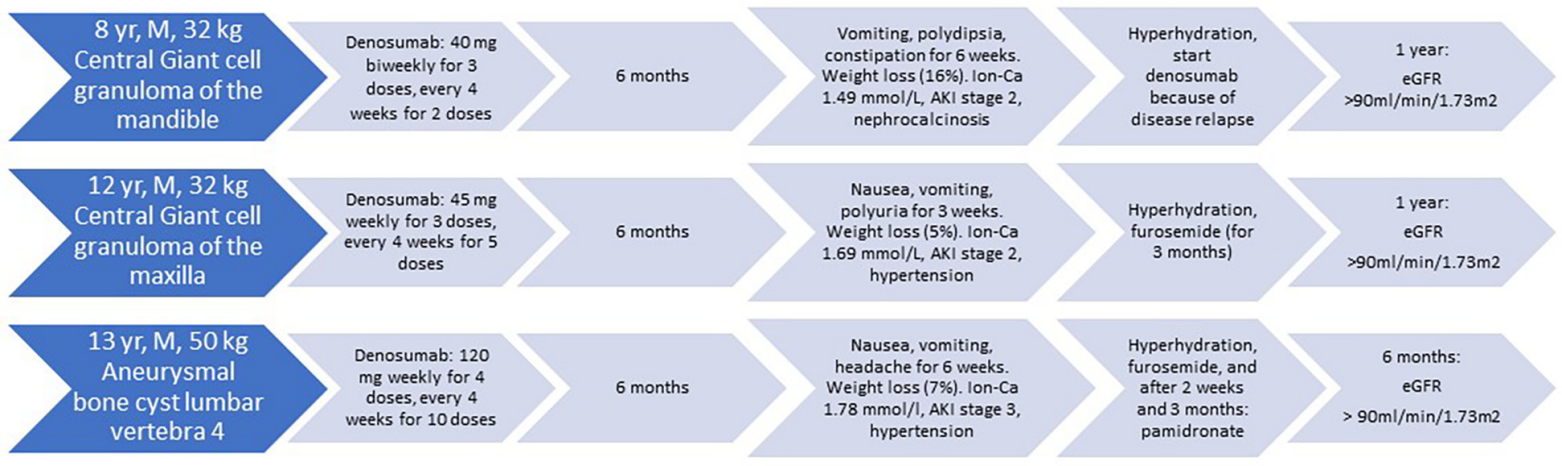
Timeline of three patients.

Three pediatric patients, aged 8, 12, and 13 years at the time of presentation, were treated with denosumab at the University Medical Center Utrecht and the Princess Máxima Center for Pediatric Oncology Utrecht, The Netherlands. A timeline with most relevant data of care is showcased in Figure 1. In summary, we describe three patients, who presented with symptoms six months after discontinuation of denosumab therapy, accompanied by severe hypercalcemia that was not driven by PTH, PTH-related peptide or calcitriol. Additionally, they exhibited AKI, nephrocalcinosis (case 1), hypertension (case 2 and 3), and hypercalciuria.

### Patient 1

An 8-year-old male with developmental delay and a biopsy-confirmed central giant cell granuloma of the mandible (CGCG) involving the region 43–45 was treated with intralesional denosumab at a dosage of 1.25 mg/kg (35 mg/m^2^) biweekly for three doses, followed by monthly administration for two additional doses. Baseline laboratory investigation showed normal serum levels of (ionized and total) calcium, phosphate, alkaline phosphatase, 25-hydroxyvitamin D and parathyroid hormone (PTH). To mitigate the risk of hypocalcemia, supplementation with calcium (500 mg/day) and vitamin D (400 IU/day) was initiated. Serum calcium concentrations were monitored prior to each denosumab injection and remained within the normal reference range throughout the treatment course. Therapy was discontinued after five doses due to clinical and radiographic evidence of tumor ossification. Surveillance for hypercalcemia was not conducted.

Six months following the final denosumab administration, the patient presented with a six-week history of vomiting, polydipsia, constipation, and a 16% reduction in body weight. Laboratory evaluation revealed hypercalcemia (ionized calcium 1.49 mmol/L; total plasma calcium unadjusted for albumin 2.81 mmol/L, 11.4 mg/dl), suppressed PTH (<1 pmol/L) and reduced 1,25-dihydroxyvitamin D (<12 pmol/L). The patient also met criteria for AKI (KDIGO stage 2) with serum creatinine elevated to 80 µmol/L from a baseline of 40 µmol/L. Urinalyses showed a fractional calcium excretion of 2.6% and a urine calcium-to-creatinine ratio of 1.16 mol/mol. Dual energy x-ray absorptiometry (DEXA) demonstrated normal bone mineral density, and kidney ultrasound revealed mild bilateral cortical nephrocalcinosis.

The patient was advised to increase oral fluid intake and received an additional dose of denosumab (40 mg) due to clinical and radiological evidence of CGCG recurrence; a repeat dose was administered four months later. Subsequent serum calcium levels remained within normal limits. At one-year follow-up, estimated glomerular filtration rate (eGFR) was preserved (>90 ml/min/1.73 m^2^).

### Patient 2

A 12-year-old male was diagnosed with biopsy-confirmed CGCG of the maxilla, involving the region 22–23. The patient was treated with intralesional denosumab at a dosage of 1.4 mg/kg (38 mg/m^2^) administered weekly for three doses, followed by monthly administration for five additional doses. Baseline laboratory investigations were within normal limits. Throughout the course of treatment, the patient received daily supplementation with calcium (500 mg/day) and vitamin D (400IE). Serial monitoring of serum calcium demonstrated normal levels of both total and ionized calcium. The treatment was discontinued following near-complete remission of the lesion. No follow-up monitoring for hypercalcemia was performed.

Six months after cessation of denosumab therapy, the patient presented with symptoms, including nausea, vomiting, polyuria and a 5% reduction in body weight persisting over a three-week period. Stage 1 hypertension was noted on examination. Laboratory investigations revealed significant hypercalcemia (ionized calcium 1.69 mmol/L, total calcium unadjusted for albumin 3.44 mmol/L, 13.8 mg/dl), suppressed PTH (<1 pmol/L) and 1,25-dihydroxyvitamin D (<12 pmol/L) and PTH-related peptide was not high (0.9 pmol/L). Acute kidney injury was diagnosed (KDIGO stage 2), with serum creatinine levels rising from a baseline of 35 µmol/L to double the initial value. Electrocardiogram showed a slightly shortened QTc-interval. Urinalyses demonstrated a fractional calcium excretion of 2.9% and a urine calcium-to-creatinine ratio of 1.4 mol/mol. DEXA revealed normal bone mineral density. Kidney ultrasonography was not performed. The patient was advised to increase oral fluid intake to at least 2 L per day and was initiated on furosemide 20 mg twice daily. After one week of treatment, symptomatic improvement was observed, and ionized calcium almost normalized to 1.42 mmol/L, prompting discontinuation of furosemide. Subsequent clinical and biochemical evaluations remained within normal limits. At one-year-follow-up there was a normal eGFR of >90 ml/min/1,73 m^2^.

### Patient 3

A 13-year-old male was diagnosed with a biopsy-confirmed aneurysmal bone cyst (ABC) located in the fourth lumbar vertebra. Treatment consisted of subcutaneous administration of denosumab at a dose of 2.5 mg/kg (75 mg/m^2^) weekly for four doses, followed by monthly administration for an additional ten doses. Baseline laboratory investigations were within normal limits. During treatment, the patient received daily supplementation with calcium (500 mg/day) and vitamin D (400IE). Serial monitoring showed consistently normal levels of both total and ionized serum calcium. Denosumab therapy was discontinued upon radiological remission of the lesion. Monthly surveillance for the rebound phenomenon showed no abnormalities during the first five months following treatment cessation and was subsequently discontinued.

Six months following the discontinuation of denosumab therapy, the patient presented with symptoms including nausea, vomiting, headache, and a 7% reduction in body weight persisting over a six-week period. On physical examination, stage 1 hypertension was noted. Laboratory investigations revealed marked hypercalcemia with an ionized calcium concentration of 1.78 mmol/L and a total calcium unadjusted for albumin level of 3.76 mmol/L (15.3 mg/dl). PTH was suppressed (<1 pmol/L), 1,25-dihydroxyvitamin D was undetectable (<12 pmol/L) and PTH-related peptide was not high (0.9 pmol/L). Acute kidney injury was diagnosed (KDIGO stage 3), with serum creatinine elevated to 204 µmol/L. Urinalyses demonstrated a fractional calcium excretion of 10.4% and a urine calcium-to-creatinine ratio of 2.2 mol/mol. Kidney ultrasonography showed no evidence of nephrocalcinosis. The patient was admitted to the hospital and initiated on intravenous 0.9% sodium chloride at 2 L per day in addition to oral intake. Pharmacological treatment included furosemide 60 mg twice daily and amlodipine 10 mg once daily. After one week, intravenous hydration was transitioned to nasogastric fluid administration, and the patient was discharged. One week post-discharge, there was no clinical improvement, and ionized calcium remained elevated at 1.75 mmol/L. Consequently, a single dose of pamidronate (1 mg/kg) was administered. Within one week, both clinical status and laboratory values improved, allowing for discontinuation of furosemide, amlodipine and hyperhydration. Three months later, the patient presented again with polyuria and weight loss. Ionized calcium was measured at 1.41, prompting administration of an additional dose of pamidronate (90 mg), which resulted in clinical and biochemical normalization. Subsequent follow-up revealed sustained clinical and laboratory stability. At six months post-intervention, kidney function was normal with an eGFR of >90 ml/min/1,73 m^2^.

## Analysis of literature

We performed a query of the Food and Drug Administration Adverse Event Reporting System (FAERS) database, which includes global adverse event data, to denosumab with a more detailed look at “renal and urinary disorders” in patients <18 years of age. In addition, in January 2025 a systematic literature search was conducted across multiple databases including Pubmed, Embase, Web of Science, and the Cochrane Library using the keywords “denosumab” AND “kidney OR renal” AND “child”. As rebound hypercalcemia was the most reported complication, we also conducted a search with “denosumab” AND “hypercalcemia” AND “child” as search terms. Bibliographical data were imported into EndNote 21 (Clarivate, London, UK), and duplicates were removed. The title and abstract of available publications were reviewed by one author (GB), and publications that do not describe cases with kidney complications of denosumab in children were excluded.

We identified 25 cases of kidney complications following treatment with denosumab (Supplementary Table S1). All observed kidney complications were associated with “rebound hypercalcemia”, occurring at a median of 5 months after discontinuation of denosumab. AKI was the most frequently reported complication (*n* = 21). This was followed by hypercalciuria (*n* = 10) and nephrocalcinosis (*n* = 7). There were no reports about chronic kidney disease. The management strategies employed for these patients predominantly included hyperhydration (*n* = 23) and the administration of a bisphosphonate (*n* = 18). Additionally, 14 patients received furosemide, 7 were treated with calcitonin, in 7 denosumab was re-administered, and 5 received corticosteroid treatment.

## Discussion

In this case series and literature review we present data of kidney complications associated with rebound hypercalcemia after discontinuation of denosumab. The observed kidney sequelae encompassed AKI, hypercalciuria, nephrocalcinosis, and hypertension. Given the increase in application of denosumab and the high incidence (about one-third) of rebound hypercalcemia reported in the pediatric population, clinicians should maintain increased awareness of potential kidney complications.

Denosumab-related kidney complications were exclusively associated with rebound hypercalcemia. Hypercalcemia affects kidney function through several mechanisms: reduced glomerular filtration rate due to vasoconstriction of the afferent arterioles, volume depletion resulting from natriuresis, nephrocalcinosis, direct tubular injury, and in long term the formation of kidney stones (8). In our cases 1 and 2, kidney function improved directly after start of hyperhydration. In case 3, kidney function showed improvement following five days of hyperhydration, although kidney function did not return to baseline and serum calcium levels remained elevated. In this patient there may have been acute tubular injury due to prolonged ischemia. There were no reports about kidney complications during denosumab treatment, in contrast to a previously documented adult case with an interstitial nephritis (9). Additionally, no reports of long-term reduced kidney function were identified, although there are several cases with nephrocalcinosis.

The literature has proposed several preventive strategies to mitigate the risk of rebound hypercalcemia following denosumab discontinuation, such as body weight-based adjustment of the dose, a weaning regimen, and the short-term administration of bisphosphonates (10, 11). However, consensus-based recommendations are lacking.

The management of hypercalcemia is primarily determined by its severity and associated symptoms. Initial interventions typically involve hyperhydration with normal (0.9%) saline for rehydrating the patient and diluting serum calcium concentration, coupled with the administration of loop diuretics to further enhance urinary calcium excretion. Subsequent treatment modalities may include the use of calcitonin, corticosteroids, bisphosphonates, and re-administration of low dose denosumab. Calcitonin is an effective agent for the initial management of severe hypercalcemia. However, itsefficacy is transient due to tachyphylaxis and should be combined with agents that have a more sustained effect (e.g., bisphosphonates) (12). Moreover, in 2012, the European Medicines Agency (EMA) completed a review of the benefits and risks of calcitonin-containing medicines, concluding that there was evidence of a small increased risk of cancer with long-term use of these medicines (13). Corticosteroids exhibit particular utility in conditions characterized by increased synthesis of 1,25-dihydroxyvitamin D3, such as sarcoidosis. In the cases of rebound hypercalcemia, corticosteroid therapy proved insufficient for optimal hypercalcemia control. Bisphosphonates are efficacious in reducing bone turnover and managing hypercalcemia, with a maximum effect in two to four days. Bisphosphonates have a long track record of safety, especially pamidronate (0.5–1.0 mg/kg) which is used most in children (12). Nevertheless, their use is associated with potential adverse effects, including flu-like symptoms, electrolyte disturbances, and AKI, and its use is contraindicated in case of severe kidney impairment and bisphosphonate allergy. In the current literature review, one patient exhibit acute interstitial nephritis after zoledronate (14) and another case developed symptomatic hypocalcemia and hypomagnesemia (muscular cramp and prolonged QTc) (15). The re-administration of (low dose) denosumab appears to be highly effective in managing hypercalcemia. In an observational study of patients with hypercalcemia of malignancy, no difference in response rate was observed between denosumab and iv bisphosphonates; however, the risk of subsequent hypocalcemia was lower with bisphosphonates (16). Based on trials in patients with cancer and bone metastases, the 2023 Endocrine Society Clinical Practice Guideline suggests denosumab over bisphosphonates for initial therapy of hypercalcemia of malignancy (17). Recent case reports showed a sustained effect of denosumab in pediatric patients with bisphosphonate refractory hypercalcemia of malignancy (18, 19), suggesting that denosumab may offer advantages over bisphosphonates. In case of rebound hypercalcemia, this approach carries the risk of precipitating a subsequent episode of rebound hypercalcemia 4–6 months later. Therefore, careful monitoring and individualized treatment strategies are essential in the management of hypercalcemia, especially in complex cases with underlying kidney dysfunction.

With an expanding clinical use of denosumab, our findings underscore the importance of educating patients and their caregivers about potential symptoms such as nausea, vomiting, polydipsia, and weight loss after discontinuation of therapy. In addition, monthly monitoring of serum calcium levels and kidney function, particularly within the 4–7-month period post-therapy cessation, is recommended to reduce the risk of kidney complications.

## Data Availability

The raw data supporting the conclusions of this article will be made available by the authors, without undue reservation.
