# Supplementary material for: Case report of hypercalcemia-related kidney complications after discontinuation of denosumab
Source: Front Pediatr. 2025 Jun 6;13:1583240. doi: 10.3389/fped.2025.1583240 (PMC12179199; doi:10.3389/fped.2025.1583240)
Supplement: Supplementary file 1 [file Table1.docx]

| **Supplemental 1.** Cases with hypercalcemia-related kidney complications after discontinuation of denosumab. | | | | | | | | | | | |
| --- | --- | --- | --- | --- | --- | --- | --- | --- | --- | --- | --- |
| **Literature reference** | **Reason for Use** | **Kidney complication** | **Sex** | **Patient Age start denosumab** | **Treatment duration (months)** | **Treatment course** | **Cumulative dose (mg)** | **occurrence time of hypercalcemia (months)** | **Peak calcium (mg/dl)** | **Initial treatment measures** | **Additional treatment measures** |
| ^1^ | Bone Giant Cell Tumour patella | AKI stage 3 | F | 10 | 24 | 120 mg weekly for 4 doses, every 4 weeks for 23 doses | 3240 | 1 | 16.6 | hyperhydration, calcitonin, bisphosphonate, | denosumab (14mg) |
| ^2^ | Osteogenesis Imperfecta type VI | hypercalciuria, nephrocalcinosis | M | 1 | 31 | 1mg/kg every 2-6 months | 11mg/kg | 2 | ion Ca 1.62 mmol/L | bisphosphonate |  |
| ^3^ | Central Giant Cell Granuloma mandible | polydipsia, nocturia, high blood pressure, hypercalciuria, AKI st3, no CKD | M | 6 | 15 | 80 mg weekly for 4 doses, every 4 weeks for 4 doses, following by a 3-month interval | 920 | 3 | 20 | hyperhydration, calcitonin | bisphosphonate |
| ^4^ | Hypercalcemia following stem cell transplantation for Autosomal recessive osteopetrosis | Hypercalciuria, nephrocalcinosis | F | 3 | 21 | Start with increasing dose to 0,25mg/kg every 4 weeks, subsequently every 8 weeks. From 9 months, dosing frequency 0,6mg/kg every 6 weeks for 12 months. Weaning schedule with introduction of zoledronic acid | NA | 3 | 15.3 | denosumab, bisphosphonate | denosumab, bisphosphonate |
| ^5^ | Bone Giant Cell Tumour Lumbosacral vertebra | AKI | F | 11 | 12 | 60 mg weekly for 4 doses, every 4 weeks for 11 doses | 900 | 3 | 15.8 | Hyperhydration, furosemide | corticosteroids |
| ^5^ | Bone Giant Cell Tumour thoracal spine | AKI, hypertension, nephrocalcinosis | M | 13 | 27 | 60 mg weekly for 4 doses, every 4 weeks for 11 doses. After 18 months again monthly infusion for 7 months. | 1320 | 3 | >16 | Hyperhydration, furosemide, corticosteroids, after precautional bisphosphonate treatment | bisphosphonate |
| ^6^ | Aneurysmal Bone Cyst sacrum | AKI stage 2, hypercalciuria | F | 10 | 25 | 60 mg weekly for 4 doses, every 4 weeks for 12 doses, and every 8 weeks for 6 doses | 1320 | 4 | 11.6 | hyperhydration, furosemide | bisphosphonate |
| ^7^ | Central Giant Cell Granuloma mandible | AKI stage 3, polyuria, polydipsia, leukocyturia (sterile), nephronmegaly, decreased corticomedullary differentiation (TIN na zeledronate). After 1 month: normal kidney function, nephrocalcinosis. | M | 13 | 12 | 100 mg weekly for 4 doses, every 4 weeks for 11 doses | 1500 | 4 | 15.4 | hyperhydration | bisphosphonate |
| ^8^ | Aneurysmal Bone Cyst pelvis | AKI stage 3, nephrocalcinosis. | M | 10 | 12 | 120 mg weekly for 4 doses, every 4 weeks for 10 doses | 1680 | 4 | 15.5 | hyperhydration, furosemide | bisphosphonate |
| ^8^ | Central Giant Cell Tumour mandible | AKI stage 2, hypercalciuria, nephrocalcinosis. After 1 month: normal kidney function | M | 14 | 12 | 100 mg weekly for 4 doses, every 4 weeks for 11 doses | 1500 | 5 | 15.4 | hyperhydration, furosemide | bisphosphonate |
| ^9^ | Bone Giant Cell Tumour sacrum | AKI, hypercalciuria | M | 10 | 14 | 120 mg weekly for 4 doses, every 4 weeks for 3 doses, after a 5-month interval again 5 doses. | 1440 | 4 | 15.2 | hyperhydration, furosemide, corticosteroids, calcitonin | bisphosphonate |
| ^10^ | Central Giant Cell Granuloma mandible | AKI | F | 10 | 5 | 70mg/m2 weekly for 5 doses, every 4 weeks 5 doses | 700mg/m2 | 5 | 13.5, ion Ca 1.78 mmol/l | denosumab, calcitonin, bisphosphonate |  |
| ^11^ | Cherubism | hypercalciuria | M | 11 | 12 | 80 mg every 4 weeks for 12 doses | 960 | 5 | 12.9 | hyperhydration, furosemide, calcitonin, bisphosphonate | bisphosphonate |
| ^12^ | Central Giant Cell Granuloma mandible | AKI | M | 11 | 14 | 70 mg/m2 weekly for 4 doses, every 4 weeks for 11 doses | 1050 mg/m2 | 5 | NA | hydration and low dose denosumab. |  |
| ^12^ | Central Giant Cell Granuloma maxilla | AKI | F | 12 | 14 | 70 mg/m2 weekly for 4 doses, every 4 weeks for 11 doses | 1050 mg/m2 | NA | NA | hydration and low dose denosumab |  |
| ^12^ | Central Giant Cell Granuloma mandible | AKI | F | 6 | 14 | 70 mg/m2 weekly for 4 doses, every 4 weeks for 11 doses | 1050 mg/m2 | NA | NA | hydration and low dose denosumab. |  |
| ^13^ | Central Giant Cell Granuloma mandible | nephrocalcinosis | M | 6 | 10 | NA | NA | 5 | 15.0 | Hyperhydration, furosemide, corticosteroids | bisphosphonate |
| ^14^ | Aneurysmal Bone Cyst cervical vertebra | AKI stage 1, hypertension | M | 8 | 12 | 70 mg/m2 weekly for 4 doses, every 4 weeks for 11 doses | 1050mg/m2 | 6 | 14.8 | hyperhydration, furosemide, bisphosphonate |  |
| ^15^ | Aneurysmal Bone Cyst tibia | AKI stage 3, hypertension, polyuria | M | 13 | 17 | 70 mg/m2 weekly for 4 doses, every 4 weeks for 6 doses, thereafter dose interval to 2-3 months | 1554 | 6 | 16.8 | hyperhydration, furosemide | calcitonin, bisphosphonate |
| ^16^ | Bone Giant Cell Tumour sacrum | AKI stage 2 | M | 14 | 16 | NA | 2200 | 6 | 13.6 | hyperhydration, bisphosphonate |  |
| ^16^ | Bone Giant Cell Tumour sacrum | AKI stage 3 | M | 15 | 46 | NA | 5520 | 7 | 12.4 | hyperhydration, calcitonin | calcitonin |
| ^17^ | Central Giant Cell Granuloma maxilla | AKI | F | 9 | 12 | 120 mg weekly for 4 doses, every 4 weeks for 8 doses | 1440 | 6 | 17.0 | Hyperhydration, furosemide, corticosteroids | bisphosphonate, corticosteroids |
| This report | Central Giant Cell Granuloma mandible | AKI stage 2, hypercalciuria, nephrocalcinosis | M | 8 | 6 | 40 mg biweekly for 3 doses, every 4 weeks for 2 doses | 200 | 6 | 11.4, ion Ca 1.49 mmol/L | hyperhydration | denosumab |
| This report | Central Giant Cell Granuloma maxilla | AKI stage 2, hypercalciuria, hypertension | M | 12 | 8 | 45 mg weekly for 3 doses, every 4 weeks for 5 doses | 360 | 6 | 13.8, ion Ca 1.69 mmol/L | hyperhydration, furosemide |  |
| This report | Aneurysmal Bone Cyst lumbar vertebra | AKI stage 3, hypercalciuria, hypertension | M | 13 | 12 | 120 mg weekly for 4 doses, every 4 weeks for 10 doses | 1680 | 6 | 15.3, ion Ca 1.78 mmol/L | hyperhydration, furosemide | bisphosphonate |

1. Gossai N, Hilgers MV, Polgreen LE, Greengard EG. Critical hypercalcemia following discontinuation of denosumab therapy for metastatic giant cell tumor of bone. *Pediatr Blood Cancer*. Jun 2015;62(6):1078-80. doi:10.1002/pbc.25393

2. Trejo P, Rauch F, Ward L. Hypercalcemia and hypercalciuria during denosumab treatment in children with osteogenesis imperfecta type VI. *J Musculoskelet Neuronal Interact*. Mar 1 2018;18(1):76-80.

3. Liu X, Xie Y, Tang J, Zhong J, Lan D. Hypercalcemia in Children Following a Discontinuation of Denosumab Therapy: A Case Report and Literature Review. *Clin Pediatr (Phila)*. Jun 2024;63(6):750-754. doi:10.1177/00099228231194427

4. Taylor-Miller T, Sivaprakasam P, Smithson SF, Steward CG, Burren CP. Challenges in long-term control of hypercalcaemia with denosumab after haematopoietic stem cell transplantation for TNFRSF11A osteoclast-poor autosomal recessive osteopetrosis. *Bone Rep*. Jun 2021;14:100738. doi:10.1016/j.bonr.2020.100738

5. Sydlik C, Durr HR, Pozza SB, Weissenbacher C, Roeb J, Schmidt H. Hypercalcaemia after treatment with denosumab in children: bisphosphonates as an option for therapy and prevention? *World J Pediatr*. Oct 2020;16(5):520-527. doi:10.1007/s12519-020-00378-w

6. Gandolfi A, Shaaban S. Denosumab-Induced Rebound Hypercalcemia Treated With Bisphosphonates in a Pediatric Patient. *JCEM Case Rep*. Sep 2023;1(5):luad133. doi:10.1210/jcemcr/luad133

7. Brijs K, Veys K, Schepers S, Segers H, Politis C. Treatment of central giant cell granuloma with denosumab: A case report of a complicated treatment course. *Pediatr Blood Cancer*. Jun 2022;69(6):e29436. doi:10.1002/pbc.29436

8. Deodati A, Fintini D, Levtchenko E, et al. Mechanisms of acute hypercalcemia in pediatric patients following the interruption of Denosumab. *J Endocrinol Invest*. Jan 2022;45(1):159-166. doi:10.1007/s40618-021-01630-4

9. Setsu N, Kobayashi E, Asano N, et al. Severe hypercalcemia following denosumab treatment in a juvenile patient. *J Bone Miner Metab*. Jan 2016;34(1):118-22. doi:10.1007/s00774-015-0677-z

10. Tallent B, Padilla RJ, McKay C, Foreman AKM, Fan Z, Blatt J. Response of Central Giant Cell Granuloma of the Jaw to Imatinib. *J Pediatr Hematol Oncol*. Jul 1 2023;45(5):278-280. doi:10.1097/MPH.0000000000002608

11. Marpuri I, Cheung C, Moke D, Rayabets-Lienhard A. Symptomatic Rebound Hypercalcemia After Denosumab Discontinuation in a Pediatric Patient With Cherubism. *J Endocr Soc* 2021;3(5 (Suppl 1)):A706-A707.

12. Choe M, Smith V, Okcu MF, et al. Treatment of central giant cell granuloma in children with denosumab. *Pediatr Blood Cancer*. Mar 2021;68(3):e28778. doi:10.1002/pbc.28778

13. Isguven P, Sabit B, Okur E, Aydin D. 10th Individual Abstracts for International Meeting of Pediatric Endocrinology: Free Communication and Poster Sessions, Abstracts. *Horm Res Paediatr*. 2017;88 Suppl 1:1-628. doi:10.1159/000481424

14. Del Sindaco G, Berlanga P, Brugieres L, et al. Mineral and Bone Consequences of High Dose Denosumab Therapy to Treat an Aneurysmal Bone Cyst, a Child Case Report. *Front Endocrinol (Lausanne)*. 2021;12:698963. doi:10.3389/fendo.2021.698963

15. Harcus M, Aldridge S, Abudu A, et al. The Efficacy of Denosumab in the Management of a Tibial Paediatric Aneurysmal Bone Cyst Compromised by Rebound Hypercalcaemia. *Case Rep Pediatr*. 2020;2020:8854441. doi:10.1155/2020/8854441

16. Uday S, Gaston CL, Rogers L, et al. Osteonecrosis of the Jaw and Rebound Hypercalcemia in Young People Treated With Denosumab for Giant Cell Tumor of Bone. *J Clin Endocrinol Metab*. Feb 1 2018;103(2):596-603. doi:10.1210/jc.2017-02025

17. Mariz B, Migliorati CA, Alves FA, et al. Successful denosumab treatment for central giant cell granuloma in a 9-year-old child. *Spec Care Dentist*. Jul 2021;41(4):519-525. doi:10.1111/scd.12588
